# Quality adjusted coverage of family planning services in low- and middle-income countries: Analysis of 33 countries using Demographic and Health Survey data

**DOI:** 10.7189/jogh.14.04125

**Published:** 2024-06-28

**Authors:** Elizabeth A Hazel, Safia S Jiwani, Abdoulaye Maïga, Gouda Roland M Mady, Emily Wilson, George Mwinnyaa, Agbessi Amouzou

**Affiliations:** Department of International Health, Johns Hopkins Bloomberg School of Public Health, Baltimore, Maryland, USA

## Abstract

**Background:**

Monitoring service quality for family planning programmes in low- and middle-income countries (LMICs) has been challenging due to data availability. Self-reported service quality from Demographic and Health Surveys (DHS) can provide additional information on quality beyond simple service contact.

**Methods:**

The DHS collects need, use and counselling for contraceptives. We used this data from 33 LMICs to develop quality-adjusted demand for modern family planning satisfied indicator (DFPSq). We compared it with the crude indicator (demand for family planning satisfied (DFPS)) and performed an equity analysis. Median, interquartile ranges (IQR) and the absolute and relative gap by country were used to describe the findings.

**Results:**

The median DFPS was 49% (IQR = 41–57%) and the median DPFSq was 19% (IQR = 14–27%). We found similar relative differences in the gap stratified by SES indicating quality was universally low. One exception is that adolescents had a higher relative gap (70%, IQR = 57–79%) compared to adults (54%, IQR = 46–68%), indicating lower quality access.

**Conclusions:**

Severe and pervasive quality gaps exist in family planning services across most LMICs. Our novel DFPSq indicator is one additional tool for monitoring access and quality of service that is critical to meet the family planning needs of women.

Despite progress in family planning programmes over the last 50 years, the number of women globally with unmet need for modern contraceptive services remains unacceptably high. In 2022, 17% or 214 million women have unmet need for services ranging from 25% in sub-Saharan Africa to 10% in Eastern and Southeast Asia [1]. Among 22 countries in sub-Saharan Africa with a Demographic and Health Survey (DHS) since 2015, the median proportion of women by country who discontinue their contraceptive method within 12 months of use is 36%, and 10% discontinue due to concerns about side effects [2]. Complete and accurate contraceptive counselling including elements of informed choice, common side effects and how to address them will reduce the number of discontinuation due to concerns about side effects [3–5].

Demand for family planning services satisfied with modern methods (DFPS) measures the proportion of women with a demand for family planning (those who want to delay or limit or avoid childbirth) who are currently using a modern contraceptive. This Sustainable Development Goal indicator reflects whether women’s and couple’s fertility preferences are being met by measuring use and need of modern contraceptives but does not reflect the quality of services received when the contraceptive was obtained [6].

Improved metrics known as effective coverage indicators measure the proportion of a population in need who receive quality health services, capturing elements of need, use, and quality [7–9]. In many low-and-middle-income countries (LMICs), there is evidence of a significant gap between coverage and effective coverage, indicating a substantial lack of quality services during contact with the health system [10,11].

Applying the cascade framework and terminology developed by Amouzou and colleagues to demand for family planning services satisfied, women in need of contraceptives must access services (‘service contact’), obtain the contraceptive (‘crude coverage’), receive the contraceptive according to quality standard (‘quality-adjusted coverage’) and continue use of the contraceptive as long as there is need (‘user-adjusted coverage’) to achieve family planning programme goals [12]. An estimate of quality-adjusted coverage for contraceptives indicates that a woman in need, accessed and received a modern contraceptive at a delivery point with sufficient evaluation and quality counselling for an informed choice to reduce discontinuation due to dissatisfaction, side effects or method failure.

Quality-adjusted coverage indicators can be created by linking household surveys that often measure contact with services with health facility assessments that measure readiness and quality of care [13]. However, linking protocols can be complex and constrained by data availability – the household and health facility surveys must be geographically and temporally aligned to be linked [14]. In some cases, need, contact, use, and quality can be derived from one data source such as in the DHS or other household surveys that rely on respondents’ reports on services received to estimate the quality of services.

Previous studies have used DHS data to estimate effective coverage for antenatal care that include reported components of care received (i.e. blood/urine sample collected or weight measured) [15,16]. Carvajal-Aguirre et al included eight postnatal interventions collected through the DHS to generate a quality postnatal care coverage indicator [11]. Other household surveys have been used to estimate quality-adjusted coverage for delivery, postnatal care of infants, and childhood immunisation [17–20]. Using the same data source to generate quality-adjusted coverage simplifies the analytical process and increases the available data for analysis.

Tracking the use of family planning services, use of service delivery points offering quality care, and measuring the coverage of family planning service accounting for quality service is a more effective way of assessing progress in reproductive health goals. We aimed to develop and apply a quality-adjusted family planning coverage indicator in LMICs) with a recent DHS.

## METHODS

As of June 2022, there were 33 DHSs with data collected between 2015 and 2019 with data sets publicly available and all the questions on contraceptive counselling included from the phase 7 core questionnaire (Annex 1 in the **Online Supplementary Document**). The majority were in the sub-Saharan Africa region (n = 19), followed by Southern Asia (n = 5) and data collection was initiated in 2015–2019 (**Table 1**). Sample size of women with family planning data collected ranged from 2778 in Armenia to 353 211 in India (Annex 1 in the **Online Supplementary Document**). The total sample across all thirty-three countries was 1.2 million women aged 15–49 years. Women over the age of 49 with data on contraceptive use were excluded. Demographic and Health Surveys are nationally representative household surveys that have been implemented in over 90 LMICs since the mid-1980s. Households are randomly sampled and typically all women ages 15–49 who reside in the household are interviewed to obtain their demographic and health information, including fertility preferences and contraceptive use.

**Table 1:** Description of the 33 countries in the analysis

Country	Survey Year (first)	Married	Urban	Adol-escent	Currently using									Used for less than 6m*	Source*		
					**None**	**Pill**	**IUD**	**Inject-ions**	**Sterilisation**	**Condoms**	**Implants**	**Other Modern**	**Tradi-tional**		**Public**	**Private**†	**Other**‡
**Caribbean **																	
Haiti	2016	63%	45%	4%	51%	3%	0%	25%	2%	13%	3%	1%	4%	71%	56%	20%	23%
**Central Asia**																	
Tajikistan	2017	99%	25%	0%	44%	4%	35%	2%	2%	8%	0%	2%	4%	79%	89%	1%	11%
**Oceania**																	
Papua New Guinea	2016	76%	14%	2%	41%	4%	1%	14%	14%	2%	15%	0%	10%	83%	95%	3%	2%
**South Eastern Asia **																	
Indonesia	2017	99%	49%	0%	14%	16%	7%	39%	5%	3%	6%	0%	9%	79%	35%	48%	17%
Philippines	2017	68%	45%	1%	24%	29%	5%	7%	11%	3%	2%	1%	20%	77%	59%	8%	33%
Timor-Leste	2016	88%	30%	1%	49%	4%	4%	23%	3%	0%	12%	1%	4%	81%	91%	8%	1%
**Southern Asia**																	
Afghanistan	2015	100%	29%	1%	52%	15%	3%	10%	4%	7%	0%	3%	6%	76%	55%	40%	5%
India	2019	96%	33%	0%	1%	7%	3%	1%	58%	14%	0%	1%	15%	81%	N/A	N/A	N/A
Maldives	2016	94%	40%	1%	63%	4%	1%	2%	9%	12%	1%	0%	7%	78%	55%	10%	36%
Nepal	2016	99%	62%	2%	31%	6%	2%	12%	27%	6%	4%	0%	13%	70%	74%	18%	9%
Pakistan	2017	100%	41%	1%	34%	3%	4%	5%	17%	18%	1%	0%	18%	77%	45%	25%	30%
**Southern Europe**																	
Albania	2017	89%	59%	1%	25%	1%	1%	0%	2%	3%	0%	0%	68%	79%	44%	56%	1%
**Sub-Saharan Africa **																	
Angola	2015	12%	76%	8%	68%	6%	0%	8%	0%	14%	1%	1%	2%	64%	45%	4%	51%
Benin	2017	64%	45%	5%	64%	3%	3%	5%	0%	5%	11%	1%	7%	74%	63%	13%	24%
Burundi	2016	68%	11%	1%	51%	3%	2%	20%	1%	3%	11%	1%	9%	74%	83%	11%	6%
Cameroon	2018	46%	61%	6%	48%	3%	2%	8%	1%	22%	5%	3%	8%	72%	31%	12%	57%
Ethiopia	2016	92%	19%	2%	38%	3%	4%	39%	1%	0%	14%	1%	1%	71%	87%	11%	2%
Gambia	2019	92%	71%	2%	55%	4%	1%	19%	1%	1%	14%	0%	4%	68%	78%	6%	16%
Guinea	2018	75%	41%	6%	60%	6%	2%	7%	1%	5%	7%	11%	1%	69%	63%	8%	28%
Liberia	2019	25%	61%	8%	51%	6%	0%	29%	0%	3%	9%	1%	2%	71%	59%	15%	27%
Malawi	2015	79%	17%	3%	25%	3%	1%	37%	14%	4%	15%	0%	1%	75%	80%	18%	3%
Mali	2018	89%	27%	5%	57%	5%	3%	14%	1%	0%	18%	0%	2%	72%	77%	10%	13%
Mauritania	2019	98%	49%	4%	69%	15%	0%	7%	0%	1%	4%	0%	3%	60%	87%	4%	9%
Nigeria	2018	80%	54%	2%	52%	4%	2%	8%	1%	9%	9%	4%	13%	74%	56%	28%	16%
Rwanda	2019	53%	18%	1%	19%	8%	3%	19%	2%	5%	35%	2%	7%	78%	90%	5%	5%
Sierra Leone	2019	59%	49%	8%	46%	10%	1%	22%	0%	1%	18%	1%	1%	73%	82%	6%	12%
South Africa	2016	27%	67%	4%	20%	8%	2%	38%	7%	20%	5%	0%	1%	77%	82%	12%	7%
Tanzania	2015	55%	38%	3%	34%	8%	1%	20%	5%	8%	11%	1%	11%	70%	65%	20%	15%
Uganda	2016	41%	26%	4%	40%	3%	2%	27%	4%	6%	9%	2%	6%	67%	60%	32%	8%
Zambia	2018	75%	44%	4%	31%	10%	1%	35%	2%	6%	12%	1%	3%	71%	90%	5%	5%
Zimbabwe	2015	80%	37%	2%	14%	48%	1%	13%	1%	8%	14%	1%	1%	76%	79%	8%	13%
**Western Asia **																	
Armenia	2015	99%	58%	0%	18%	4%	13%	0%	1%	21%	0%	1%	42%	81%	34%	3%	63%
Jordan	2017	100%	90%	0%	22%	12%	32%	1%	2%	8%	0%	2%	22%	78%	48%	34%	17%

N/A –not applicable, NGO – non-governmental organisation, IUD – intrauterine device

*Among women currently using a modern method.

†Includes all non-public, health facility-based delivery points including non-profit, for profit, NGOs, and religious.

‡Includes all informal sector delivery points including private pharmacies, shops, religious organisations, schools, friends/relatives and ‘other private’. ‘Don't know,’ ‘missing,’ and ‘other’ sources are excluded.

Women are identified in need of contraceptives if they have been sexually active in the previous 30 days, fecund, not menopausal, reported a mistimed pregnancy or birth, or are currently using any type of contraceptive, including traditional methods. Women using a contraceptive method are asked to describe the type of method. Modern methods are defined as hormonal methods (pills, injections, implant, or emergency contraceptive pills), intrauterine devices (IUDs), female or male sterilisation, female or male condoms, standard days method (SDM), lactational amenorrhea method (LAM), or other modern methods (e.g. Billings method).

**Figure 1:**
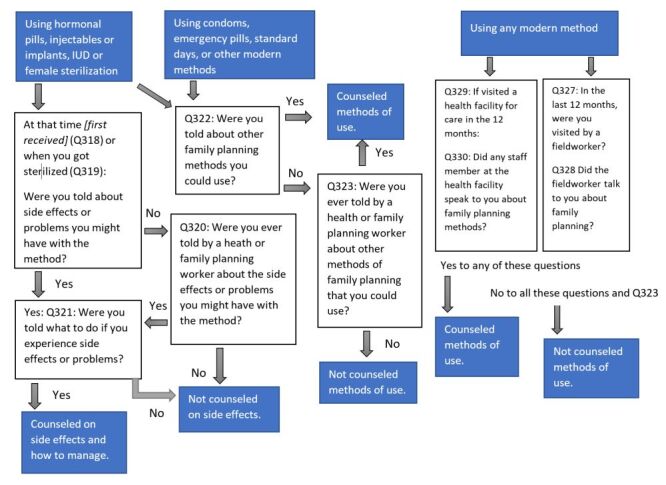
Structure of questions on contraceptive counselling from the phase 7 core questionnaire and how they are categorised for the indicator.

The method information index (MII) measures the quality of counselling received during family service prior to the start of the contraception: whether the woman was told about (1) other family planning methods, (2) the side effects of the current method, and (3) what to do if the woman experiences side effects [21]. In DHS, women using modern contraceptive methods are asked a series of questions to determine whether they were appropriately counselled. Women currently using hormonal pills, injections, implants, or IUDs were asked if they were counselled on the side effects of the method and how to manage them. **Figure 1** presents the sequence of questions on contraceptive counselling from the DHS phase seven core questionnaire. These women – and those currently using condoms, emergency contraceptive pills, SDM, LAM, or other modern methods – were asked whether they were told about other methods when they obtained their current method. All women who reported visiting a health facility or being attended by a field worker in the previous 12-month were asked whether they were counselled about family planning.

### Defining the quality-adjusted indicator

**Figure 2:**
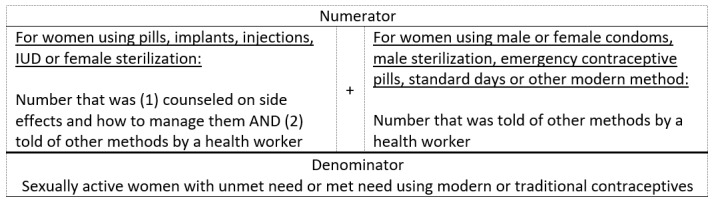
Demand for family planning satisfied using modern methods indicator definition.

The steps for calculating the DFPS indicator are described elsewhere [22]. For the quality-adjusted demand for family planning services satisfied by modern methods (DFPSq) we defined the numerator as (1) all women currently using a modern contraceptive who were counselled on other methods by a health worker at the time they received a method, or at any time in the previous 12 months and (2) women using hormonal pills, injections, implants, IUD or female sterilisation counselled on side effects and how to manage (**Figure 2**). We defined the denominator as all sexually active women with a demand for family planning (those with unmet need and women with met need using modern or traditional contraceptives). Countries that collected contraceptive use among married women only were included.

### Analysis

We conducted our analysis across countries using medians and interquartile ranges (IQR). We calculated DFPS and DFPSq indicators using the Amouzou et al. cascade framework and analysed both the absolute percentage point and relative gap between the two indicators by country and world region. We then performed a country-level analysis of DFPSq by urban-rural locality, marital status, wealth quintile, highest level of women’s education (any or no formal education), time since first use of current contraceptive (less than six months vs. six to 59 months), adolescence age of women (less than 18 years of age vs. 18–49 years of age), type of contraceptive currently used: short (SARC) or long-acting reversible contraceptive (LARC). All analyses were performed in Stata 16.1 and the script for calculating the quality-adjusted DFPS is available in Annex 2 in the **Online Supplementary Document** [23].

## RESULTS

Of the 1.2 million women in the 33 DHS data sets, 581 526 women were identified as in need of contraceptives services. Most women were married (median 80% by country). In Afghanistan, Pakistan and Jordan, only married women were asked about contraceptive use. Adolescents under the age of 18 comprised 2% of the sample by country (IQR from 0–4%). Among women who wanted to limit or delay childbirth, almost half were not using contraceptives (median = 41%, IQR = 25–52%). Injectables were the most common method by country (median = 13%, IQR = 7–23%), followed by implants (7%, IQR = 1–12%), condoms (6%, IQR = 3–9%), and traditional methods (6%, IQR = 2–11%) (**Table 1**). Among women using a modern method, most have been using it less than six months (median = 74%) and contraceptive source varied by country, but public sector was the most predominant source (median = 64%).

**Figure 3:**
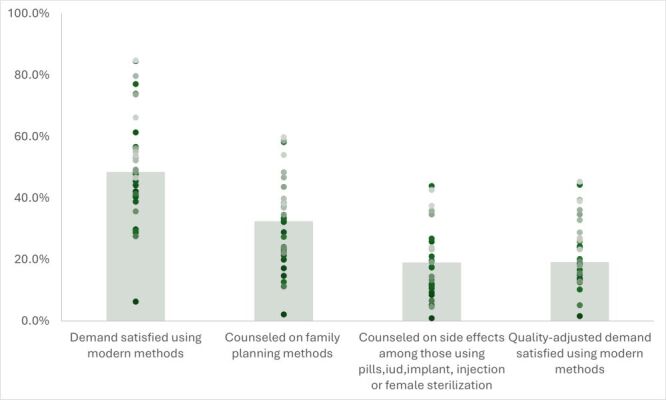
Country-level coverage cascade. (1) The proportion of women with demand for family planning satisfied using all modern methods; (2) The proportion of women with demand satisfied using any modern method who reported counselling of family planning from a health worker; (3) The proportion of women with demand for family planning satisfied using intrauterine device (IUD), female sterilisation or hormonal pills, implants, or injections who were counselled on side effects and how to manage when they received their most recent contraceptive method; (4) The proportion of women with demand for family planning satisfied using any modern methods who reported counselling on family planning and received counselling on side effects (if applicable).

The country-level DFPS median was 49% (IQR = 41–57%), ranging from 6% in Albania to 85% in Zimbabwe (**Figure 3**). The median proportion of women in need of contraceptives who were counselled on family planning was 33% (IQR = 22–38%). Among women using an IUD, female sterilisation, hormonal pills, implants or injectables 19% (IQR = 11–24%) were also counselled on the side effects and how to manage them. The DFPSq was 19% (IQR = 14–27%), ranging from 2% in Albania and 45% in Zambia and was considerably lower in all countries compared to DFPS.

**Figure 4:**
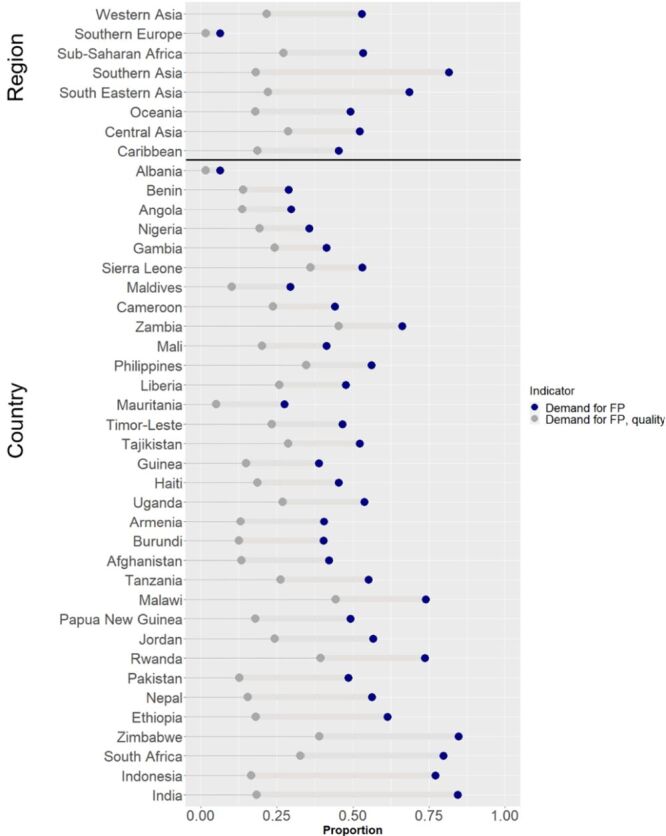
Demand for family planning satisfied using modern methods (DFPS) compared to the quality-adjusted indicator (DFPSq), global region and country (countries are sorted by the size of the gap).

**Figure 5:**
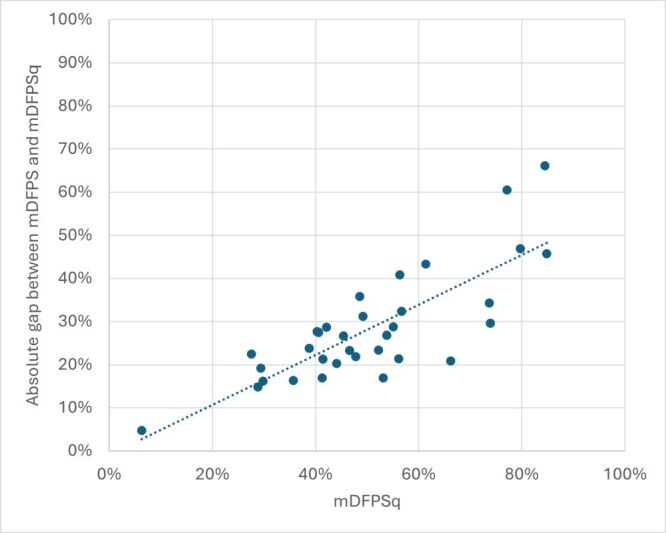
Demand for family planning satisfied using modern methods (DFPS) compared to the gap between DFPS and the quality-adjusted indicator (DFPSq) with a linear trend line.

The median absolute gap between DFPS and DFPSq was 27 percentage points (pp) (IQR = 21–32%), ranging from 5 pp in Albania and 66 pp in India (**Figure 4**). The relative median gap was 60% (IQR = 66–52%). Southern and Southeast Asia had the highest DFPS and the largest absolute gap while countries in sub-Saharan Africa had lower DFPS but a smaller absolute gap between DFPS and DFPSq (**Figure 4**). There was a positive linear relationship between DFPS and the absolute coverage gap (**Figure 5**). Countries with higher DFPS had a higher absolute gap between DFPS and DFPSq. Because of this we examined the relative gap for the equity stratification analysis.

We found higher DFPS and DFPSq for women with higher socioeconomic status (urban vs. rural, any vs. no formal education, and wealthier vs. poor), with a similar relative gap for all groups (**Table 2**, Figure S1 in the **Online Supplementary Document**). We found no difference in coverage between married and unmarried women, and adolescent girls had lower DFPS and DFPSq with a higher relative gap in coverage compared to adult women (**Table 2**, Figure S1 in the **Online Supplementary Document**).

**Table 2:** Demand for family planning satisfied using modern methods: crude and quality-adjusted indicators and the absolute and relative gap by equity

	DFPS, median (IQR) %	DFPSq, median (IQR) %	Absolute gap, median (IQR) pp	Relative gap, median (IQR) %
Locality				
*Urban*	52 (16–30)	24 (16–30)	31 (22–37)	53 (46–67)
*Rural*	48 (16–30)	18 (13–27)	25 (18–30)	55 (48–71)
Household wealth				
*Wealthiest/highest quintile*	55 (49–63)	25 (19–29)	33 (24–37)	56 (46–65)
*Poorest/lowest quintile*	40 (28–55)	16 (10–26)	22 (14–30)	58 (45–69)
Highest level of formal education completed				
*Any*	52 (44–60)	22 (17–28)	28 (22–32)	54 (47–65)
*None*	42 (35–57)	15 (11–24)	23 (17–35)	56 (46–73)
Current marital status				
*Married*	48 (38–58)	21 (14–28)	24 (18–32)	54 (45–68)
*Not married*	57 (46–68)	20 (14–29)	30 (22–42)	58 (49–71)
Women/girl age				
*Adult women/18 years or older*	49 (41–57)	20 (14–28)	26 (21–32)	54 (46–68)
*Adolescent girls/15–17 years*	30 (16–44)	8 (4–14)	19 (13–28)	70 (57–79)

DFPS – demand for family planning satisfied using modern methods, DFPSq – quality-adjusted demand for family planning satisfied using modern methods, IQR – interquartile range, pp – percentage points

The median DFPS among urban women (52%, IQR = 46–62%) was higher compared to rural women (48%, IQR = 35–57%) (**Table 2**). DFPSq was also higher among urban women (24%, IQR = 16–30%) compared to rural women (18%, IQR = 13–27%). The relative gap for rural (55%, IQR = 48–71%) and urban (53%, IQR = 46–67%) women was similar. Women from the highest quintile of household asset wealth had a higher crude coverage (55%, IQR = 49–63%) and a higher quality-adjusted coverage (25%, IQR = 19–29%) compared to women from the poorest quintile of households (crude: 40%, IQR = 28–55% and quality-adjusted: 16%, IQR = 10–26%) (**Table 2**). The relative gap was similar for the wealthiest women (56%, IQR = 46–65%) compared to the poorest women (58%, IQR = 45–69%). Women with any reported formal education had a higher crude coverage (52%, IQR = 44–60%) and a higher quality-adjusted coverage (22%, IQR = 17–28%) compared to women with no formal education (crude: 42%, IQR = 35–57% and quality-adjusted: 15%, IQR = 11–24%). The relative gap was similar for women with no education (56%, IQR = 46–73%) compared to women with any formal education (54%, IQR = 47–65%) (**Table 2**).

Unmarried women had higher DFPS (57%, 46–68%) compared to married women (48%, IQR = 38–58%) (**Table 2**). However, the quality-adjusted DFPS (median: 21%, IQR = 14–28% for married and 20%, IQR = 14–29% for unmarried) and the relative gap (54%, IQR = 44.7–68.1% for married and 57.8%, IQR = 49–71% for unmarried) were similar. The three countries where only married women were asked about contraceptive use were excluded from this stratification by marital status.

Adult women 18 years of age and older had higher crude and quality-adjusted DFPS (crude = 49%, IQR = 41–57% and quality-adjusted: 20%, IQR = 14–28%) compared to adolescent girls (crude = 30%, IQR = 16–44% and quality-adjusted: 8%, IQR = 4–14%) (**Table 2**). The relative gap for adolescent girls was higher 70% (IQR = 57–79%) compared to adult women (54%, IQR = 46–68%). Armenia, Maldives, and Tajikistan were excluded from this stratified analysis since the sample did not include adolescents.

Among women using a modern method, we found DFPSq was higher for contraceptives first sourced in the public sector (median by country = 55%, IQR = 38–65%), compared to the private (47%, IQR = 34–62%) or the informal sector including private pharmacies (38%, IQR = 31–46%) although this varied widely by country (Figure S2 in the **Online Supplementary Document**). For instance, in Nigeria, DFPSq for public sector source was much higher compared to private or informal, while in Indonesia, the DFPSq was uniformly low regardless of the source. We did not analyse the gap between DFPS and DFPSq since the source data were categorical.

## DISCUSSION

In this study, we developed a quality-adjusted coverage indicator for family planning and applied it to 33 LMICs to generate national and equity stratified estimates and measured the coverage-quality gap. This is the first study to develop and apply a quality-adjusted family planning coverage indicator using only DHS.

Similar to previous studies looking at reproductive, maternal, newborn and child health, we found a significant gap between the quality-adjusted and crude coverage indicators for demand for family planning satisfied for almost all countries [11]. We found countries with higher levels of DFPS had a higher gap between DFPS and DFPSq. For instance, India and Indonesia have DFPS greater than 75% but the DFPSq is below 20%, similar to the DFPSq for Nigeria with a much lower DFPS (36%). Countries with smaller coverage gaps, like Benin (15 pp) and Albania (5 pp) also had lower DFPS (14 and 2% respectively).

Women who were wealthier, living in urban areas, with any formal education, and 18 years or older had a higher crude and quality-adjusted coverage compared to poorer women, living in rural areas who received no formal education, and less than 18 (respectively). However, the relative gap between crude and quality-adjusted coverage was the same by wealth quintile, education, and urban/rural residence indicating the level of reported counselling quality did not vary between these groups and was generally low across. However, the median relative gap for adolescents was higher compared to adults, indicating adolescents had a differentially lower access to complete counselling.

The fact that quality of care was found to be associated with health inequities, has been shown elsewhere. A recent review found seven studies in LMICs that stratified effective coverage of reproductive, maternal, newborn and child health by wealth quintile and in all cases, wealthier women had higher effective coverage of maternal and child health services compared to poorer women [10]. Another study looking at contact and content of antenatal care as reported by DHS respondents, found gaps in reported contact and completeness of care, and also inequities in the quality-adjusted coverage by women’s education, rural locality, and household wealth [16,24]. However, none of these studies presented the gap between crude coverage and quality-adjusted coverage by equity.

There is evidence in the literature of equity differences in counselling quality. Studies in Mexico and Kenya found lower levels of counselling quality among younger women and the Kenyan study found lower levels among women with no education and using short-term contraceptives [25,26]. We also found lower levels of reported counselling among adolescents but not by education. Educational access and its relationship to socioeconomic status differs by country context and in most countries, women with no education did not have differential access. However, in a majority of countries, adolescent girls had lower access.

In general, we found women who sourced their contraceptives from the public sector had higher DFPSq, although this varied by country (Figure S1 in the **Online Supplementary Document**). Previous systematic literature reviews comparing levels of quality of health care in public vs. private sector in LMICs had contradictory findings, although differing methodologies were used [27–29]. This study supports that public sector facilities had higher levels of counselling quality for family planning compared to the private sector, but a more in-depth analysis at the country level would be required to explain this finding. In this study, we pooled the country data together to further examine this pattern. The public sector was the predominant source for both urban (56%) and rural (75%) women, but more urban women used private (24%) and informal sector (20%) compared to rural women (16% private and 9% informal) (Annex 3 in the **Online Supplementary Document**). Women from wealthier households sourced their contraceptives from the private and informal sector. Adolescent girls sourced more from the informal sector and adult women sourced more from the private sector (Annex 3 in the **Online Supplementary Document**). Since adolescent girls used the informal sector more, this might account for the lower counselling quality they reported.

The main strength of this study is the scope of its application. Given the large amount of data available, we were able to apply this indicator in 33 countries to examine its performance. Although we did not perform a trend analysis, DHSs are implemented approximately every five years allowing countries to track effective coverage for family planning over time. Another strength is that the DHSs collect the MII indicator which measures whether the woman was told about (1) other family planning methods, (2) the side effects of the current method, and (3) what to do if the woman experiences side effects and has been linked to reduced method-related discontinuation. Women in need of contraceptives and who were counselled on all three items were more likely to continue their current contraceptive method over a 12-month period in Uganda and Pakistan [30]. Another study in Kenya found that women in need of contraceptive services, receiving even just one of the three counselling items, were less likely to discontinue their method [3].

Another strength of this study was that we could capture counselling for women using contraceptives not obtained through the formal health system. It is important to measure this in settings where a significant proportion of women access contraceptives in shops, private pharmacies, schools or elsewhere. The DHS asks all women about whether they received family planning counselling through a field worker or when visiting a health facility for other care. If a woman is using a method such as condoms and received this counselling, this is considered quality counselling from the health system.

The main limitation is that counselling was self-reported. It was not possible to assess the accuracy of the reporting and whether (1) the counselling received was correct; or (2) whether the counselling was understood. A study in Pakistan and Uganda asked the three MII questions and then a series of follow-up questions asking the women to describe specific counselling items [31]. Some of the women who reported counselling, described incorrect information, particularly around side effects (i.e. incorrectly report the side effects of the method they received). The authors found this discrepancy related to lower formal education, poorer household wealth, higher parity, higher age, and side effects counselling (side effects being the most complex of the three counselling components in the MII) [31]. Ideally, a clinically trained assessor would observe family planning sessions to determine whether the appropriate counselling was given. We assume women are reporting correctly on the counselling components and referring to counselling received while obtaining the current method, not on previous knowledge obtained elsewhere. Finally, this indicator does not include men.

Because the counselling questions are asked for the current method, we were not able to directly link the DFPSq indicator to an outcome such as contraceptive discontinuation as collected by the DHS contraceptive calendar module. However quality counselling is positively associated with method continuation, we can assume there is an association with impact on unmet need [30]. However, in current DHS protocols, only three counselling questions are asked. One study suggests adding a fourth question on method switching better captures quality counselling associated with continued use for women in need of contraceptive services [32].

## CONCLUSIONS

This novel indicator allows countries to monitor both contact and quality of family planning programmes, using DHS data, publicly available and widely implemented in many low- and middle-income countries. The indicator is simple to calculate and does not include analytically intensive methods or assumptions about service access involved in linking protocols. We found a large gap in the reported use of contraceptives and the quality-adjusted use among women in need of family planning services. However, the relative gap did not vary by socioeconomic status, except by adolescent age, indicating that quality is universally low. Monitoring the use and quality of counselling through the health systems is critical to meet the contraceptives needs of women and couples. This indicator is an additional tool to help improve service quality monitoring.

## Additional material


Online Supplementary Document
