# Supplementary material for: Quality adjusted coverage of family planning services in low- and middle-income countries: Analysis of 33 countries using Demographic and Health Survey data
Source: J Glob Health. 2024 Jun 28;14:04125. doi: 10.7189/jogh.14.04125 (PMC11211968; doi:10.7189/jogh.14.04125)
Supplement: Online Supplementary Document [file jogh-14-04125-s001.pdf]

Annex 1: Mapping of eligible DHSs for inclusion

Country/Year	Phase	Dates of Fieldwork	No of women with family planning data	Status
Afghanistan	DHS-VII	06/2015- 02/2016	13,172	Included
Albania	DHS-VII	09/2017- 12/2017	4,551	Included
Angola	DHS-VII	11/2015- 02/2016	5,694	Included
Armenia	DHS-VII	12/2015- 04/2016	2,778	Included
Benin	DHS-VII	11/2017- 02/2018	6,469	Included
Burundi	DHS-VII	10/2016- 02/2017	6,130	Included
Cameroon	DHS-VII	06/2018- 12/2018	5,210	Included
Ethiopia	DHS-VII	01/2016- 06/2016	5,312	Included
Gambia	DHS-VIII	11/2019- 03/2020	3,618	Included
Guinea	DHS-VII	02/2018- 06/2018	3,153	Included
Haiti	DHS-VII	11/2016- 04/2017	7,133	Included
India	DHS-VII	6/2019 - 4/2021	353,211*	Included
Indonesia	DHS-VII	07/2017- 09/2017	25,170	Included
Jordan	DHS-VII	10/2017- 01/2018	8,882	Included
Liberia	DHS-VII	10/2019- 02/2020	4,547	Included
Malawi	DHS-VII	10/2015- 02/2016	14,866	Included
Maldives	DHS-VII	03/2016- 11/2017	3,030	Included
Mali	DHS-VII	08/2018- 11/2018	3,565	Included
Mauritania	DHS-VII	11/2019 - 4/2020	4,547	Included
Nepal	DHS-VII	06/2016- 01/2017	7,655	Included
Nigeria	DHS-VII	08/2018- 12/2018	12,243	Included
Pakistan	DHS-VII	11/2017- 04/2018	7,461	Included
Papua New Guinea	DHS-VII	10/2016- 12/2018	7,206	Included
Philippines	DHS-VII	08/2017- 10/2017	11,202	Included
Rwanda	DHS-VIII	11/2019- 07/2020	6,877	Included
Sierra Leone	DHS-VII	05/2019- 08/2019	6,879	Included
South Africa	DHS-VII	06/2016- 11/2016	5,033	Included
Tajikistan	DHS-VII	08/2017- 11/2017	3,946	Included
Tanzania	DHS-VII	08/2015- 02/2016	6,158	Included
Timor-Leste	DHS-VII	09/2016- 12/2016	3,866	Included
Uganda	DHS-VII	06/2016- 12/2016	9,320	Included
Zambia	DHS-VII	07/2018- 01/2019	6,978	Included
Zimbabwe	DHS-VII	07/2015- 12/2015	5,664	Included
Bangladesh	DHS-VII	10/2017- 03/2018		Exclude - missing v3a03, v3a06, v393a
Colombia	DHS-VII	02/2015- 03/2016		Exclude - missing v3a03 or v3a06

Cambodia	DHS-VII	06/2014- 12/2014		Exclude - missing v393a
Chad	DHS-VII	10/2014- 04/2015		Exclude - missing v393a
Ghana	DHS-VII	09/2014- 12/2014		Exclude - missing v393a
Guatemala	DHS-VII	10/2014- 06/2015		Exclude - missing v393a
Kenya	DHS-VII	05/2014- 10/2014		Exclude - missing v393a
Lesotho	DHS-VII	09/2014- 12/2014		Exclude - missing v393a
Myanmar	DHS-VII	12/2015- 07/2016		Exclude - missing v393a
Rwanda	DHS-VII	11/2014- 04/2015		Exclude - missing v393a

* This was the subset of the entire sample that was interviewed on family planning

Annex 2: STATA script for calculating mDFPS and mDFPSq

https://github.com/countdownto2030/DFPSq

Annex figure 1: Quality-adjusted demand for family planning satisfied (DFPSq) stratified by (A) urban and rural locality, (B) Wealth quintile, poorest versus the wealthiest households, (C) Adolescent age of the women (Less than 18 years of age), (D) Marital status and (E) Women's highest level of education: any or no formal education.

A. Urban and rural

B. Household wealth, richest versus

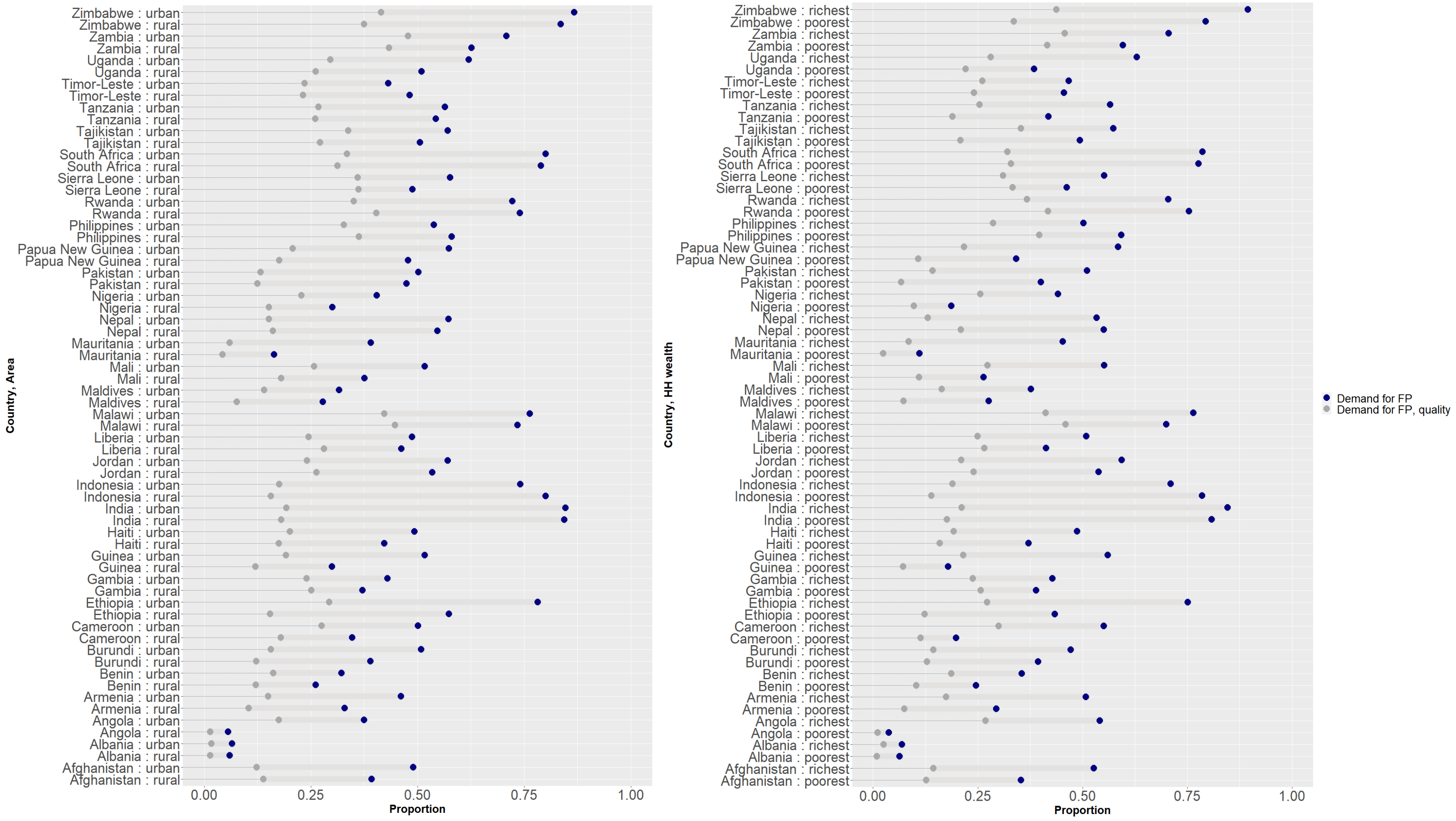

C. Adult versus adolescent

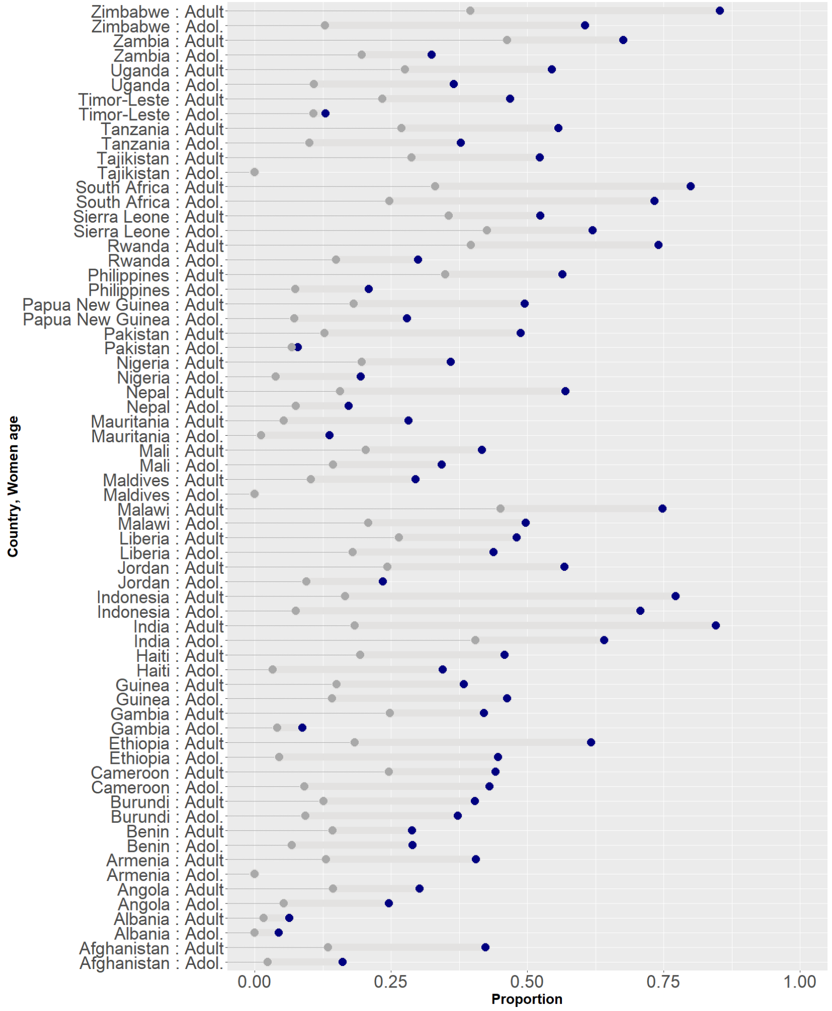

D. Marital

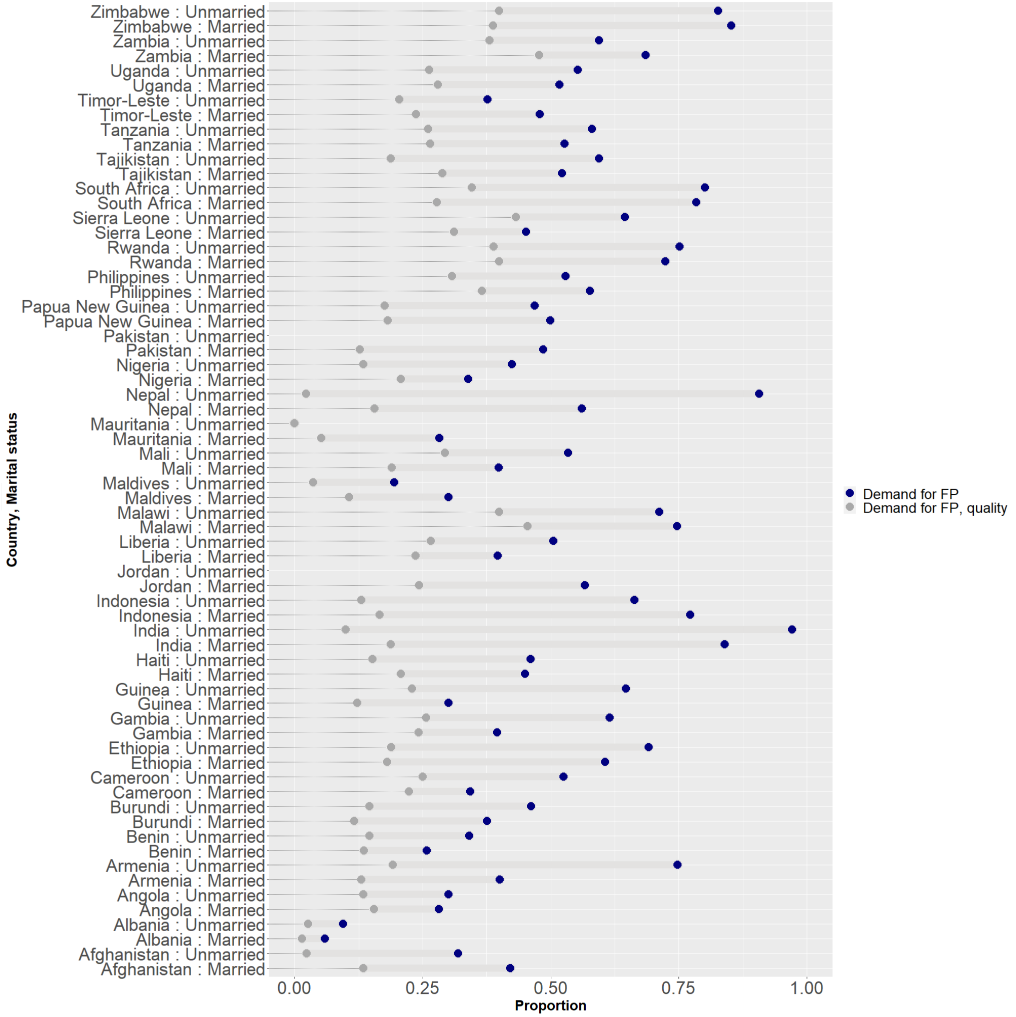

E. Highest level of formal education

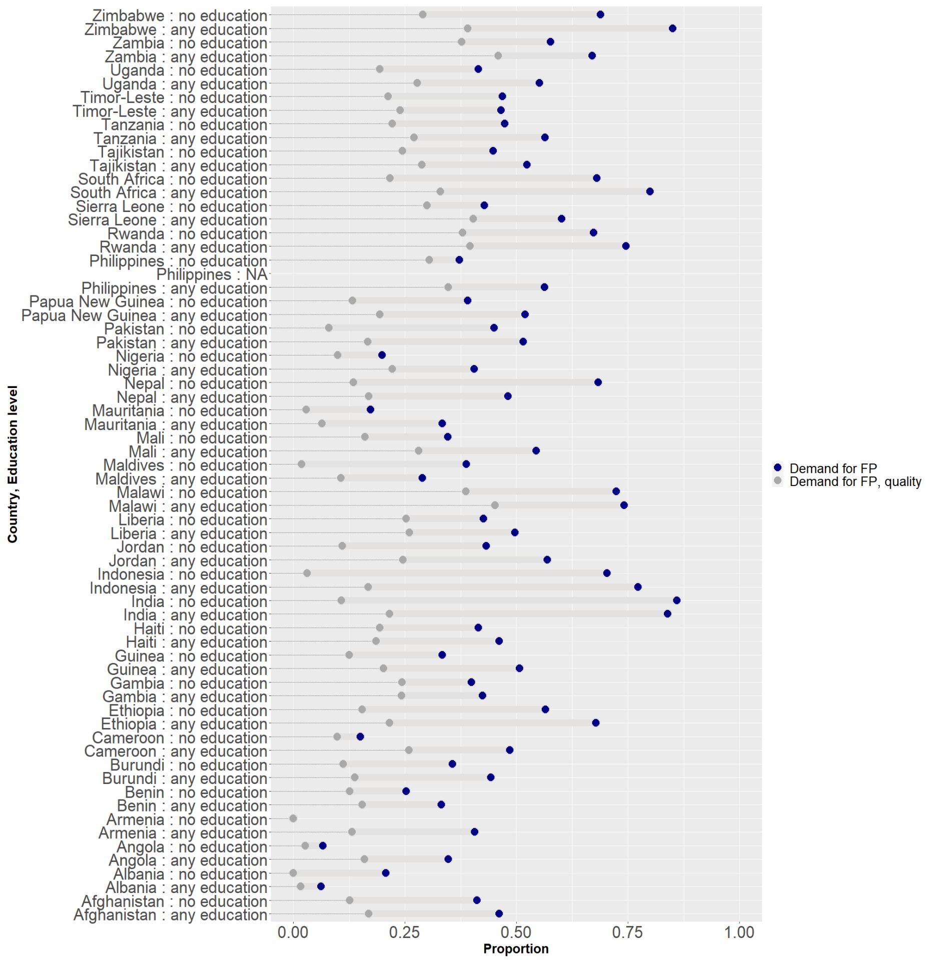

Annex figure 2: Quality-adjusted demand for family planning satisfied (DFPSq) stratified by contraceptive source: public sector, private sector (includes non-profit and for-profit facilities) and Other (informal sector including private pharmacies).

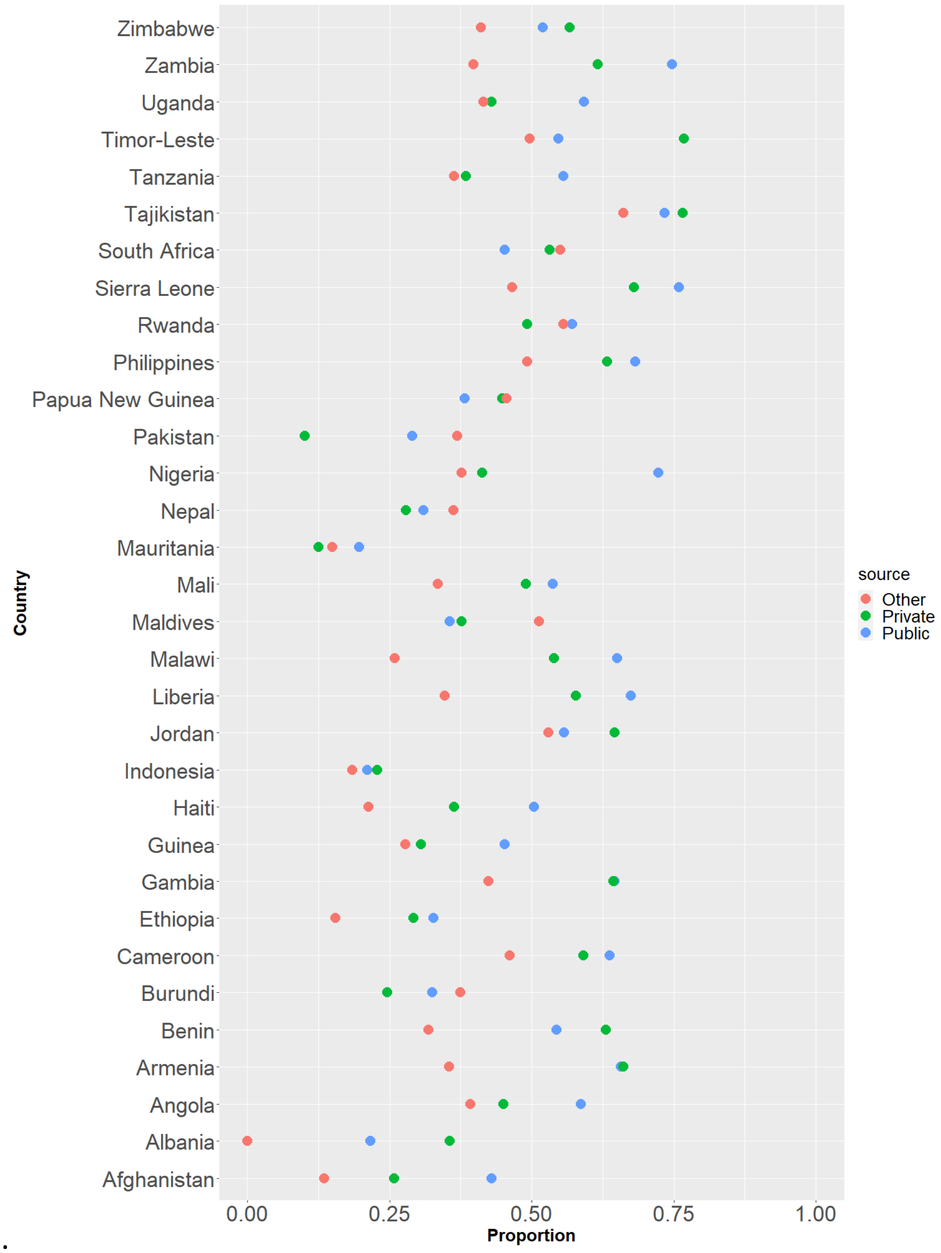

.

Annex 3. Source of contraceptives by women age, household wealth quintile (poorest and wealthiest quintile) and locality: public sector, private sector (includes non-profit and for-profit facilities) and Informal (including private pharmacies), pooled country data.

	**Public**<br>**% (n)**	**Private**<br>**% (n)**	**Informal**<br>**% (n)**
**Adolescent**	60.29 (1,298)	11.19 (241)	28.52 (614)
**Adult**	66.85 (72,151)	19.56 (21,108)	13.59 (14,667)
**Poorest**	78.55 (15,471)	13.94 (2,745)	7.51 (1,480)
**Richest**	53.28 (12,710)	25.92 (6,184)	20.80 (4,963)
**Rural**	74.86 (47,550)	16.05 (10,194)	9.09 (5,776)
**Urban**	55.63 (25,899)	23.96 (11,155)	20.41 (9,505)
